# Weaning U.S. food-animals off antimicrobials: What can we learn from state- and city-level policies?

**DOI:** 10.1371/journal.pone.0282315

**Published:** 2023-03-15

**Authors:** Scarlet S. Bliss, Maya Homsy King, Miriam I. Bermejo, Andrew Nguyen, Heather K. Amato, Jay P. Graham

**Affiliations:** 1 UC Berkeley School of Public Health, Berkeley, California, United States of America; 2 UC Davis, Davis, California, United States of America; 3 UC San Diego, San Diego, California, United States of America; Ross University School of Veterinary Medicine, SAINT KITTS AND NEVIS

## Abstract

Antimicrobials are widely used worldwide in food animal production for controlling and preventing disease and for improving feed conversion efficiency and growth promotion. Inappropriate use of antimicrobials in animal agriculture has the potential to promote antimicrobial resistance, which represents a threat to human, animal, and environmental health. State and municipal policies in the United States have recently been implemented to improve antimicrobial use and reporting in this sector. This study analyzed the implementation of two state-level policies (California (CA) and Maryland (MD)) and a city-level policy in San Francisco (SF), California that aimed to reduce the use of antimicrobials in food-animals and increase transparency of antimicrobial use. A qualitative analysis was based on in-depth interviews with key informants (KIs) (n = 19) who had direct experience implementing or working in the context of these sub-national policies. Interviews were recorded and transcriptions were analyzed independently by two researchers using a three-stage, grounded theory coding procedure. This study identified four major findings, including 1) vague language on antimicrobial use within policies reduces policy effectiveness; 2) the lack of reporting by producers challenges the ability to evaluate the impact of the policies on actual use; 3) diverse stakeholders need to be involved in order to develop a more effective policy; and 4) funding should be linked to the policy to provide for reporting and data analysis. This analysis provides insights on the successes and failures of existing policies and serves to inform future sub-national policies aiming to improve the judicious use of antimicrobials in food-animals.

## Introduction

Antimicrobial resistance (AMR) is a growing risk to human health globally, threatening a century of medical progress in treating bacterial infections. Trends show that the prevalence of AMR is rising in many common pathogens, such as *Escherichia coli*, *Klebsiella pneumoniae*, and *Staphylococcus aureus*, and by 2050, an estimated 10 million deaths due to AMR per year could potentially be expected [1–3]. Antimicrobial resistant bacterial infections are estimated to result in 2.8 million infections and 35,000 deaths in the U.S. per year, and cost $20 billion to treat these more complex infections [4, 5]. Globally, infectious disease models estimate that 4.9 million deaths were associated with bacterial resistant to antimicrobials in 2019 [6].

Although the human impacts of AMR are felt most acutely in healthcare settings, antimicrobial use and the spread of AMR are not just a healthcare issue. One Health issues, like AMR, demonstrate human health is inextricably linked to the health of animals and the environment, and in the case of antimicrobial use in the agricultural industry, the food chain and environmental pathways connect human and animal health directly [7–10]. In crop agriculture, eleven classes of antimicrobials are used on crops across the globe, which enter environmental reservoirs through routine spraying [11]. In animal agriculture, a substantial share of U.S. antimicrobial use is devoted to raising animals for human consumption (food animals), and an estimated 90% used in animal health are excreted unmetabolized in manure [5, 11, 12]. The Natural Resources Defense Council (NRDC) estimates that 65% of all antimicrobials sold annually are for use in food animals [13]. Of those sold, 54% are medically important antimicrobials (i.e., from the same drug classes used to treat humans) [14]. The continued use of antimicrobials in U.S. food animal production creates a significant and consistent selection pressure on the microbiota in the gastrointestinal tract of food-animals that have been shown to spread through the food supply [12, 15, 16]. An increasing prevalence of AMR in the food supply and in consumers of those products can increase the risk of infection and increase the risk that those infections will be untreatable [17].

Currently, human infections in the U.S. cost around $55 billion dollars per year–with an estimated $35 billion due to indirect costs [4]. Despite this economic impact in the healthcare sector, the clinical pipeline for antimicrobials has few new drugs in development, and the majority of new therapeutics are modifications of existing ones that may only provide short-term solutions [18, 19]. Investment in replenishing the drug development pipeline to stay ahead of the threat of AMR is one component, but attention must be made to reduction of antimicrobial use, the promotion of judicious prescribing practices, and the prevention of AMR spread between the agricultural sector and humans [2, 6, 20].

**Fig 1:**
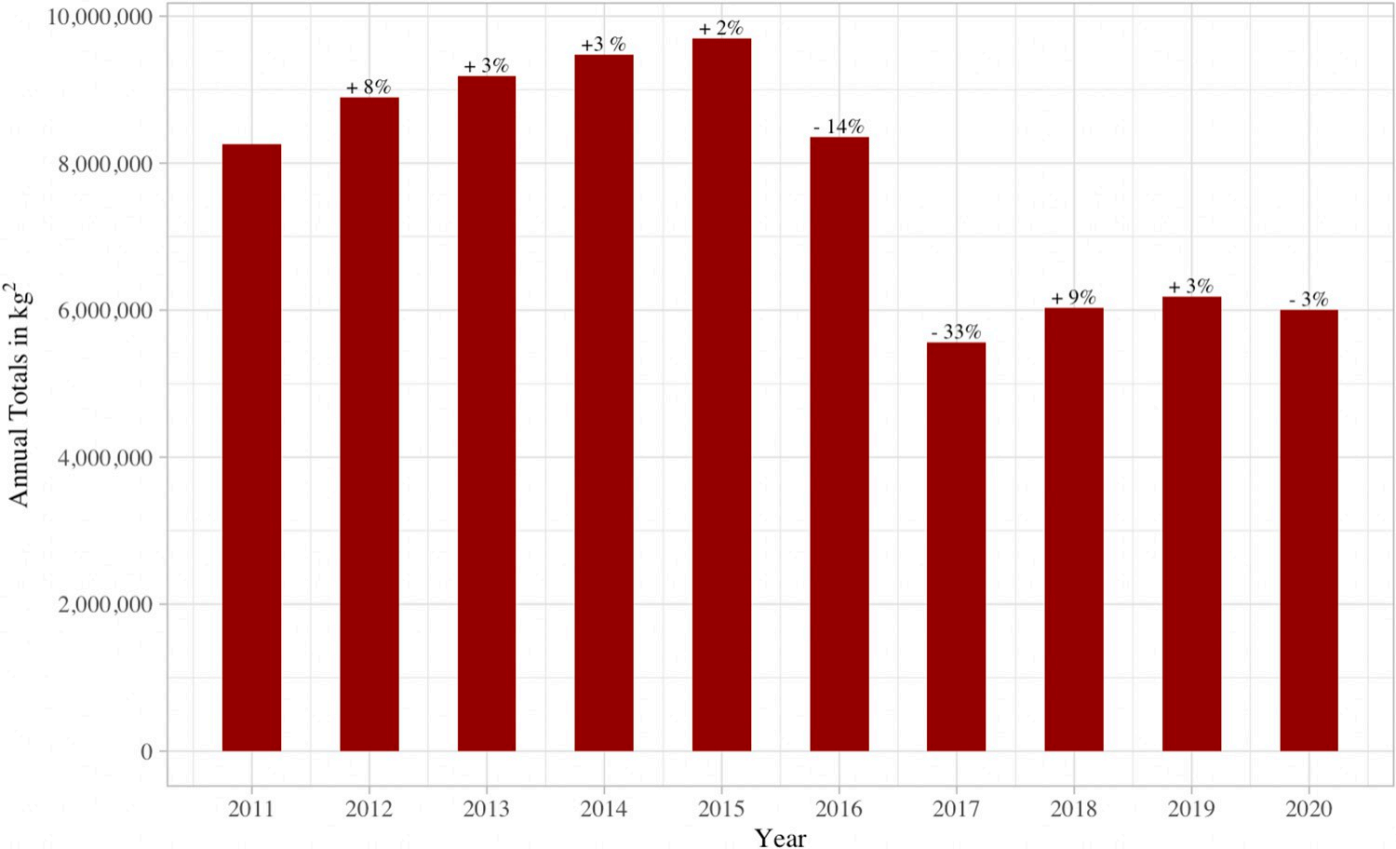
Percent annual change in sales of medically important antimicrobial drugs approved for use in food-producing animals in the United States: 2011–2020. Data from 2020 Summary Report on Antimicrobials Sold or Distributed for Use in Food-Producing Animals [14].

The U.S. Food and Drug Administration (FDA) responded to this threat in 2013 and developed national-level Guidance for Industry (GFI) regarding antimicrobial use for food-producing animals, GFI #213 [21]. The policy requires that sponsors of antimicrobials in feed and water work with the FDA to voluntarily withdraw approval of antimicrobials for productivity purposes (e.g., growth promotion), and voluntarily change antimicrobial use from over-the-counter (OTC) to either veterinary feed directive (VFD—requiring veterinarian oversight of antimicrobials used in feed) or prescription. Implementation of GFI #213 was completed in December 2016. From 2015 to 2017, antimicrobial sales for food animal production were reduced by 43%. Research suggests that there was no economic impact to meat production, and livestock producers engaged more heavily with their veterinarians to implement better management strategies [22]. Fig 1 highlights the trends in antimicrobial sales from 2011–2020.

One ongoing critique of GFI #213 is that it has not resolved antimicrobial labeling issues; most antimicrobial labels still do not specify duration of use dosage and antimicrobial regime [23]. Though an additional GFI (#263) was passed in 2021 encouraging antimicrobial producers to label medically important antimicrobials as prescription-only drugs (bringing them under veterinary oversight), advocates are encouraging the FDA to create more specific antimicrobial use reduction goals, prohibit use for disease prevention (i.e., non-therapeutic use), control the duration of use, and develop better antimicrobial use data collection and reporting [24, 25].

In the absence of further federal action, cities and states have begun to pass more aggressive regulations addressing antimicrobial use in the animal agricultural sector and/or mandating more rigorous antimicrobial use data collection and reporting (Table 1). In California, Senate Bill (SB) 27, effective January 1, 2018, authorized veterinarians to administer, dispense, or prescribe medically important antimicrobials when necessary, but prohibits administration of “a medically important antimicrobial drug in a regular pattern [26]. Maryland’s 2017 Keep Antibiotics Effective Act, which was clarified with an additional bill passed in 2019, restricts the use of antimicrobials in livestock and swine that are in good health and requires farmers and veterinarians to report antimicrobial use [27]. The City of San Francisco passed Ordinance No. 204–17 in 2017, requiring grocers in San Francisco with at least 25 total locations to report the use of antimicrobials in meat and poultry products to the Department of the Environment [28]. Policies in New York, Illinois, Oregon and Pennsylvania have been brought to the State Senates but are yet to be passed [29–32] (Table 1).

**Table 1:** U.S. sub-national policies regarding the judicious use of antimicrobials in animal agriculture.

Policy No.	State	Date filed	Date enacted		Objectives
Senate Bill (SB)-27 [26]^1^	California	10/10/15		1/01/18	*Senate Bill 27*: • Reduce overuse and misuse of medically important antimicrobials • Inhibit prescription of medically important antimicrobials^1^ for non-therapeutic uses • Reduce duration of prescriptions of antimicrobials to 1 year • Mandate continuing education program for veterinarians on judicious use of antimicrobials every 4 years • Compel the Department of Food and Agriculture to request VFDs^2^ and gather antimicrobial sales and usage data
SB-422 [33]	Maryland	5/27/17		1/01/18	*Keep Antibiotics Effective Act*: • Prohibit administration of medically important antimicrobials in feed or water to cattle, swine, or poultry unless administered via a veterinarian • Mandate reporting of annual use of medically important antimicrobials by veterinarians • Set duration limit of 21 consecutive days of antimicrobial treatment • Prohibits administration of medically important antimicrobials to dairy cattle entering a dry cycle^3^. *Clarification to the Keep Antibiotics Effective Act: • Adds and clarifies definitions to key terms including “Administered in a regular pattern,” “Control of the spread of disease or infection,” “Elevated risk,” “Prophylaxis,” and “Treat a disease or infection.” • Provided an exemption to cattle farms with herd sizes fewer than 300 cattle • Required veterinarians to report annual use of medically important antimicrobials, in addition to producers
SB-471/HB-652 [34]		4/3/19*		5/25/2019*	
No. 204–17 [35]	San Francisco, California	10/24/17		11/23/17	*Antibiotic Use in Food Animals Ordinance*: • Requires grocery store chains with at least 25 locations, one or more of which in San Francisco, to report antimicrobials used in meat products to the Department of the Environment • Reports must include the reason, certification, and amount of antimicrobial use, and whether antimicrobials are medically important • Failure to report can lead to fines incurred jointly and severally to grocers and/or producers
SB-920 [32]	Oregon	3/9/2015		*Not enacted (in committee)*	• Prohibits administration or provision of medically important antimicrobials to food-producing animals for non-therapeutic purposes • Mandates concentrated animal feeding operations to annually report administration of medically important antimicrobials • Allows for private cause of action, i.e. enables individuals to report producers who do not adhere to requirements
SB-246 [30]	Pennsylvania	1/27/2017		*Not enacted (session sine die)*	• Prohibits use of certain antimicrobial agents in agriculture in nontherapeutic amounts or as growth promoters • Gives department of health authority to designate additional antimicrobial agents that may not be administered and develop new regulations mandating annual reporting on use of antimicrobial agents in animals
SB-3429 [29]	Illinois	1/9/2019		*Not enacted (session sine die)*	• Restricts medically important antimicrobial use to that which is prescribed by a veterinarian, and only for a specified period • Veterinarian must have completed a farm visit within the last 6 months. Duration of antimicrobial use cannot exceed 21 days, and records of start and end dates must be recorded • Large, concentrated animal feeding operations (defined by the U.S. Environmental Protection Agency) must file annual reports containing specified antimicrobial use information required by the Department of Public Health
SB-3115 [31]	New York	1/27/2021		*Not enacted (in committee)*	• Prohibits non-therapeutic use of antimicrobial agents in food animals • Prohibits sale or transport of food products derived from animals who have been exposed to non-therapeutic use of antimicrobial agents within the state • Violation would lead to a class A misdemeanor

^1^“Medically important antimicrobial drug” means an antimicrobial drug listed in Appendix A of the federal Food and Drug Administration’s Guidance for Industry #152, including critically important, highly important, highly important, and important antimicrobial drugs.

^2^ Veterinary Feed Directives: a VFD drug is intended for use in animal feeds, and use of the VFD drug is allowed only under the supervision of a licensed veterinarian.

^3^Except in specific cases.

Limited research to date has looked at the implementation challenges, and successes, associated with sub-national policies in the United States agricultural sector aimed at reducing antimicrobial use. In this study, we investigated the recently enacted sub-national policies in the U.S. that aim to reduce the use of antimicrobials in food-animal production through key informant (KI) interviews. The aims of this analysis were to: 1) identify key challenges observed in writing and enacting the policies, 2) describe the impacts of their implementation, and 3) make recommendations for other states and cities that aim to reduce antimicrobial use in their jurisdiction.

## Methods

### Recruitment

The research team identified a range of key informants, including: 1) organizations advocating for improved management of antimicrobial use, 2) individuals involved in the passing of the state- and city-level policies, 3) individuals in research or academic fields with subject-area expertise on antimicrobial use policy, and 4) individuals working in the agricultural sector under one of the three policies (e.g., veterinarians). These key informants (KIs) were selected via purposive sampling based on their personal or professional expertise or direct experience with the policies. Participants provided written informed consent prior to each respective individual conversation. Participants both involved in the writing and enacting of the policy and those working directly under the implemented policy (e.g., veterinarians) were recruited to contribute information on one or multiple of the Maryland, California, and San Francisco policies.

### Data collection

Nineteen KIs contributed 22 interviews (three KIs with subject area expertise on two of the policies completed two interviews each). KIs came from veterinary, nongovernmental (NGO)/non-profit, research/academia, and law fields, and they are summarized in Table 2. Key informant interviews (KIIs) were approximately 30 minutes in length. A semi-structured interview guide was used for all interviews, with state-specific adaptations (Interview guides are available in S1 File). Interviews were conducted via videoconference and subsequently transcribed. The California and Maryland policy interviews began with 5 rating questions (0–10 scale) asking KIs to evaluate the policy on its success or failure by different metrics and its impact; Fig 1 summarizes these ratings. Maryland policy interviews asked participants to respond according to the newest version of the policy, passed in 2019.

**Table 2:** Summary of key informants (KIs) interviewed for this study.

State	Total No.	Key Informant ID	Profession category
California	8	KI1^1^	Veterinarian
		KI2^2^	Veterinarian
		KI3	Researcher/academia
		KI4	Veterinarian/academia
		KI5	Researcher/academia
		KI6	Veterinarian
		KI7^1^	NGO^3^/non-profit
		KI8^1^	Veterinarian/academia
Maryland	7	KI1^1^	Veterinarian
		KI9	Non-profit & healthcare
		KI10	NGO/non-profit
		KI11	Researcher/academia & healthcare
		KI7^1^	NGO/non-profit
		KI12	Veterinarian
		KI13	Veterinarian
San Francisco	6	KI14	Government
		KI15	NGO/non-profit
		KI16	NGO/non-profit
		KI17	NGO/non-profit; involved in drafting policy
		KI18	NGO/non-profit; involved in drafting policy
		KI8^1^	Veterinarian/academia
National	1	KI19	Non-profit & academia
Illinois^3^	2	KI20	Veterinarian
		KI21^4^	NGO/non-profit

^1^Key informant (KI) contributed information to more than one policy

^2^Interview included two veterinarians who responded together (coded as one key informant)

^3^Non-governmental organization

^4^Excluded from analysis, as policy has yet to be enacted

### Data analysis

We applied a grounded theory approach to text analysis. Grounded theory, an inductive and iterative method of data analysis, builds a conceptual understanding of qualitative data through a series of coding steps on qualitative data [22]. This method is frequently used in KII analysis as it enables the sorting, synthesis, and conceptualization of large amounts of qualitative data [22]. For the purposes of this study, it allowed us to consolidate actionable information from experts with differing opinions involved in the writing and implementation of these policies. Two authors (SB, MHK) coded the transcriptions independently, using a codebook generated by all authors, and subsequently compared the results of their independent coding. Where discrepancies arose, a third author (JG) was brought in to evaluate the results, which were then discussed by all three authors (SB, MHK, JG) until consensus was reached. Though findings were analyzed predominantly through this inductive approach, a priori themes can be identified in the content of our semi-structured interview questions (see S1 File for script). These include: 1) reasons for success or failure of the policy, 2) positive or negative effects of policy implementation, and 3) considerations for future antimicrobial use policy.

We used the qualitative research software Atlas.ti (Version 22.0.2) to conduct the analysis [36]. Open coding was used to identify underlying concepts, axial coding was used to develop categories and properties, and selective coding was used to identify the key themes of the collective qualitative data [24]. Multiple readings and development of codes and themes were conducted by two researchers and compared to ensure reproducibility of analyses [37].

### Ethics approval

The study was approved by the IRB (Protocol # 2021-07-14474) by the Office for Protection of Human Subjects (OPHS) of the University of California, Berkeley.

## Results

### Ratings

Prior to identifying the emergent themes from the interviews, we examined KI responses to a series of semi-quantitative rating questions, particularly comparing CA and MD policies. The questions asked KIs to rate the policy on a scale from 0 to 10, 0 being completely ineffective and 10 being completely effective, on different criteria.

**Fig 2:**
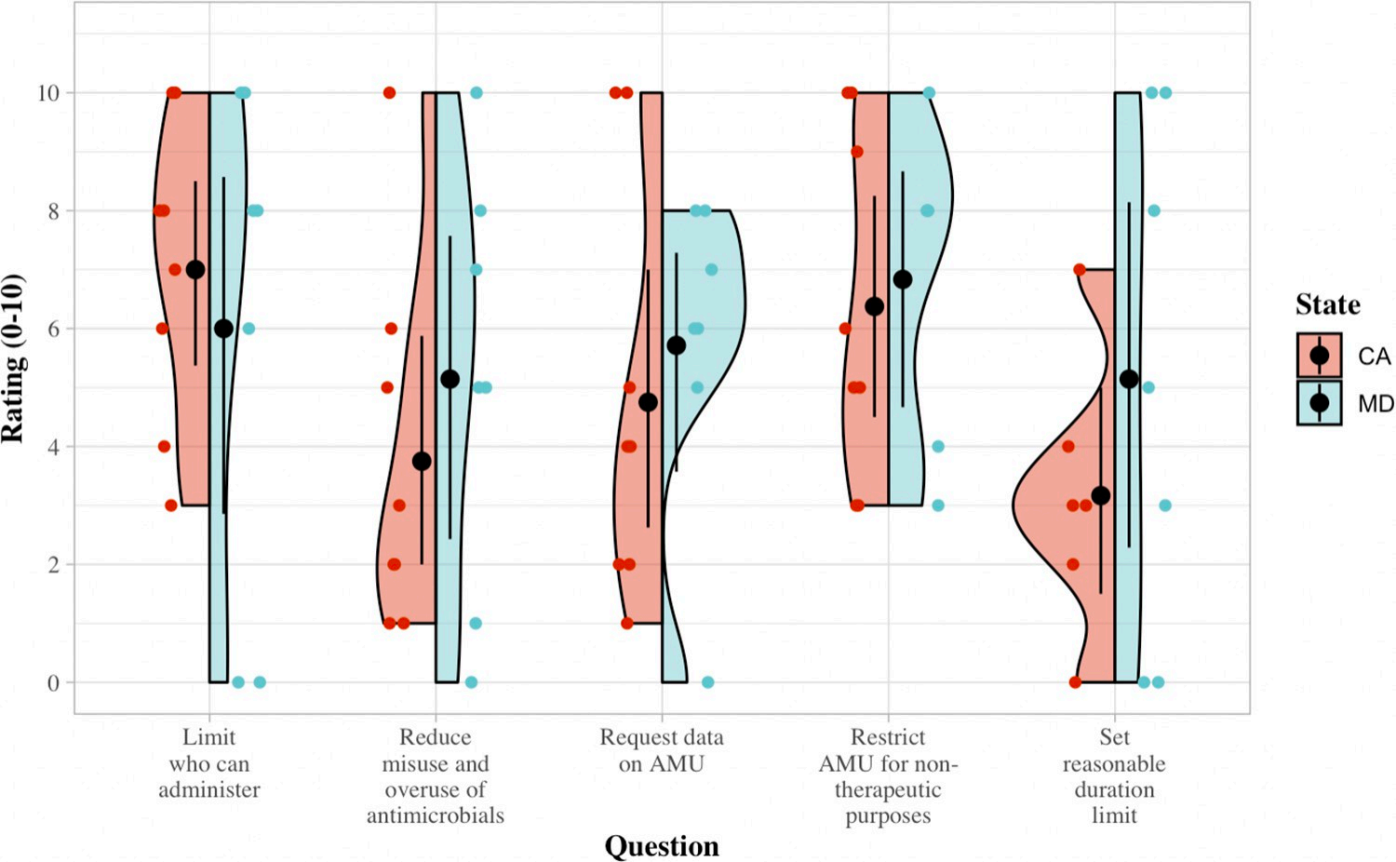
Key informant ratings of California (red) and Maryland (blue) policies on five metrics, displaying median and interquartile range (IQR) of participant responses.

On average, the MD policy received higher ratings across performance criteria questions (Fig 2). These differences were echoed by findings from the semi-structured interview portion of each KII. The only question for which the CA policy received higher ratings was Q2 (Did the policy limit who can administer, dispense, or prescribe medically important antimicrobials to livestock?). Based on responses during in-depth interviews, many KIs concurred that federal policy on VFDs already established prescribing regulations on antimicrobials, so these ratings are likely a reflection of the fact that the state-level policies just reiterated existing law (GFI #213). Additionally, ratings for each question and both policies ranged significantly, likely reflecting the range in opinions based on the profession of the KIs. Those responsible for writing and lobbying for the policy, for example (non-profit, legal counsel, and public health professionals), often rated policies as more effective than veterinarians.

### Themes

Based on the in-depth interviews, four themes were identified in the analysis of policy evaluations by KIs. These included: 1) data reporting challenges; 2) clarity of policy language; 3) the role of stakeholder engagement and resistance in policy implementation; and 4) the political level at which antimicrobial use regulations should be enforced (city, state, or federal). These themes highlight the differences between the policies in question, and findings provide insight for policymakers to inform more effective legislative decisions in the future regarding judicious use of antimicrobials.

#### Data collection, analysis, and reporting challenges

KIs with expertise in the CA policy noted the lack of data available to evaluate its effectiveness as a weakness of the bill. In its writing or implementation, California did not propose resources that could be used for analyzing collected antimicrobial use data, nor did they clarify data reporting requirements for producers. KI3 noted that though the California Department of Food & Agriculture (CDFA) has the authority to collect sales and usage data, they have not made it publicly available. Additionally, KIs commented that regarding data, the data collection provisions of the CA policy have been implemented in the most lenient way possible, translating to an opt-in approach to data reporting that is highly vulnerable to selection bias (and thus likely underestimates sales and usage).

KI 5 notes that, though three years of data reporting is too soon to tell,

*“The bill could be a false promise in that the public probably believe something has changed*, *but it’s unclear from a scientific perspective if much has changed in the state of California around antibiotic use in livestock feed*.*”* (KI 5, CA)

The combination of ambiguous data reporting language in the policy and a lack of strict implementation could be giving rise to a policy that placates the public, while having little real impact, according to KI5. CDFA data from 2017 supports this opinion: from 2017–2019, VFD orders for antimicrobials in the state have increased, not decreased [38].

The MD policy clarification, enacted a year later, added a reporting requirement for veterinarians of all antimicrobial prescriptions and clarified the policy’s language in regards to when and in what cases antimicrobials can be prescribed. KI3 comments that the first version of the MD policy was very close to the CA bill, “almost to the letter” (KI3), but the 2019 modification to the policy required quarterly antimicrobial use collection from producers, as well as charged the Animal Health Program of the Department of Agriculture to implement the data collection and reporting on all antimicrobial use in food-animals. According to KI9, the 2019 update to the MD policy showed that it is possible to collect antimicrobial use data, indicating a successful implementation of the policy. However, KIs agree that at the time of their interviews, it is too early to truly assess the success of the policy in reducing antimicrobial use by animal producers in Maryland.

Two data challenges mentioned by KIs for each policy but emphasized from Maryland interviewees were acquisition of funding for the analytics team and the complexity or incompleteness of data collected across producers. The MD policy, similar to the CA policy and the SF Ordinance, does not include support for data analysis and reporting. In Maryland’s case, the State Veterinarian’s office hired an analyst out of their own budget; however, KIs agreed that funding must accompany future policies if adequate data reporting is to occur.

KI13 commented on the complexity of different VFD and antimicrobial use data reporting formats, saying:

*“That’s a lot of work to get 50 states to agree*. *In the meantime*, *Global VetLink just went and did it…it’s just a matter of bringing in the modern age*. *We all want this big data*, *big information*, *but in order to really make it efficient*, *you need it to come electronically*. *And we have one foot in both doors*.*”* (KI 13, MD)

While the FDA has not mandated all antimicrobial use data be reported in one particular format, producers and veterinarians reported their usage data in a range of formats that makes consolidation for reporting and analysis difficult. Maryland has partnered with Global VetLink, a platform aiming to streamline animal health compliance documentation, to collect antimicrobial data without needing to contact each producer individually. KIs 9 and 10 agreed that a standardized process that is free and user-friendly will make antimicrobial use data reporting possible at larger scale, and the Maryland policy is developing an example of how this can be achieved.

For the San Francisco Ordinance, the objective with data reporting was to promote transparency of antimicrobial use in the meat and poultry industry. KI 17 explained that those who were involved in passing this Ordinance worked hard to come up with the text, think through challenges, and build out the infrastructure for collecting antimicrobial use information in a database. There was also consensus among KIs that it is too soon to tell if this Ordinance has changed antimicrobial use practices.

The challenge regarding data for San Francisco has been in who is responsible to report antimicrobial use to the city. KI 17 notes:

*“Why don’t we just require the producers to report directly to the city*? *Having the grocers be kind of the middle entity is more complicated*. *You have this trust issue… and the grocers are the endpoint that interacts with consumers*. *But bringing their brand and reputation into the mix and using their contracting power and leverage as an access point to consumers we think is a really important part of this Ordinance*. *And what makes it a potentially strong lever for local entities to play a role in changing industry practices over time*.*‬”*(KI 17, SF)

Two key points were raised–first, that, according to KIs, grocers worry the responsibility of reporting antimicrobials in their meat products will disincentivize consumer purchases, when producers should be the ones required to report. Second, KI 17 highlighted the potential impact a city-level policy can have nationally when the industry subject to regulation crosses city and state borders, as many animal producers do.

#### Clarity of policy language

KIs emphasized that there was a need for clearer language on antimicrobial use, duration of use, and data reporting in the bills. Although many mentioned that there was increased data availability and transparency on antimicrobial use resulting from the bills, it was common to hear that this was not sufficient, and that a significant reason for this was that requirements were not explicitly laid out in the language of the policy.

*“Either the fault lies with them and an erroneous interpretation of the bill which some stakeholders assert that they just did not interpret it the way it was supposed to be interpreted*. *Or you could fault the writers of the bill and say ‘should have written it tighter and really forced their hand’*. *So maybe it’s a mix of both*.*”* (KI 3, CA)

Specific improvements mentioned by key informants included making many definitions and situations clearer, such as identifying which stakeholders are responsible for what part of the implementation of the policy. KI 5 mentions that the lack of division of responsibility led to different interpretations of the bill.

*“Disagreement among stakeholders in terms of who was responsible for the implementation and which groups […] kind of the priorities of this bill and even interpretation of the bill by different groups became very problematic*.*”* (KI 5, CA)

Defining what constitutes ‘medically important’ antimicrobials, and ‘routine uses’ of antimicrobials was also mentioned as a shortcoming of the Maryland and California bills, as current wording of the bill does not specify lists of these medications and does not indicate which uses are considered routine. KI 7 also emphasizes that preventive uses of antimicrobials should be clearly mentioned and prohibited under the bills.

*“The most significant challenge for both Maryland and California was having super clear definitions […] Antibiotics should not be used to prevent disease*, *so they should only be used to treat sick animals or if need be*, *to control a verified disease outbreak on as few animals as possible at that time*. *So those definitions are very important and very challenging to make clear in both bills*.*”* (KI 7, MD/CA)

Several KIs raised concerns regarding antimicrobial dispensation. KI 7 mentioned the lack of wording on who specifically is allowed to administer antimicrobials, and in what capacity, as well as when it is appropriate to use ‘medically important’ antimicrobials on animals.

KI 2 mentioned that the FDA is currently drafting guidelines of the duration of the use of certain antimicrobials that do not have durations on their labels, and although they expressed support of this endeavor, one respondent expressed that the draft being written still allows substantial flexibility in the duration of use (KI 19).

Multiple respondents indicated the need for a tracking or enforcement system to be included in future FDA policy. This was mentioned by multiple respondents with the goal of increasing surveillance of AR, data sharing, and animal health monitoring.

*“Having an online system or cloud-based system that makes it simple and easy for the recording entities to give their data so that you’re not combing through a lot of excel sheets*.*”* (KI 14, SF)

*“All 50 states of course have different ways they want to do things—so in terms of just having an electronic certificate that everybody could agree to*, *there’s actually been years of work with all 50 states agreeing on what exact data HTML requirements are for that*. *And that’s a lot of work to get 50 states to agree*.*”* (KI 13, MD)

KIs also mentioned the importance of having funding to implement such a bill, with many responses indicating that the bill, or parts of the bills were underfunded. As mentioned in the data reporting section, administrative funding for data reporting was found to be lacking, with KI 14 explaining that San Francisco has had to work off grants to fund their data reporting.

*“They should charge a fee for the reporting*. *We have no budget for this*, *so we’re doing it on grants and work orders from our Department of Public Health*. *Figuring out a funding mechanism for implementation will be really important*.*”* (KI 14, SF)

#### Role of stakeholder engagement and resistance in policy implementation

KIs identified several stakeholders who had influence over the policy’s passing, implementation, and effectiveness. These included farmers and meat producers, political actors (such as governors and the Department of Agriculture), veterinarians, and consumers.

Producers and pharmaceutical companies were predominantly mentioned as a hindrance to passing and implementing the bill. Many KIs described producer and pharmaceutical groups working together to combat the bill.

*“All of the big producer groups*, *so the pork producer groups*, *the bovine producer groups*, *the poultry producer groups*, *they all aligned with one another*, *though they don’t always agree on things*, *and the animal pharmaceutical industry all lined up against it*.*”* (KI 1, CA)

Broadly, producers were described as uncooperative and unwilling to provide the data required by the bill. KIs described large producers as ‘powerful’, ‘intimidating’ (KI 15), and having a ‘bent towards secrecy’ (KI 18).

Specifically, beef and pork groups were mentioned as the most uncooperative to the SF Ordinance, while poultry producers were described as highly cooperative, setting the example for going antimicrobial-free. What must be noted though, is that several KIs indicated that the shift in the poultry industry happened more-or-less independently of the policies being passed, and that it has been effective as a marketing strategy for poultry.

*“The chicken industry has made a lot of progress in reducing antibiotic use*, *and they’re also more transparent about their practices*.*”* (KI 18, SF)

*“There are places where it’s a market advantage—like poultry producers are being more transparent-because that’s a selling point over beef*, *right*? *… These producers are going to drag their feet until they’re forced to make a change*, *and then they will be putting ‘no antibiotics used’ on their products like they wanted to do it the entire time*.*”* (KI 16, SF)

Several KIs expressed the view that veterinarians should have been more a part of the writing of the bill and the decisions made on the specifics of the bill. One KI (KI 3) mentioned that the Veterinary Medical Association should have been more involved in decisions regarding the preventive use of antimicrobials, however the Veterinary Medical Association was mentioned numerous times as a body that tried to prevent the bill from passing.

*“Know that the Department of Ag and the state Veterinary Medical Association will not likely back a bill and will actively oppose it*.*”* (KI 1, CA/MD)

One veterinarian described an instance where they offered their expertise for the Maryland bill but were rejected.

*“Work with sympathetic and knowledgeable veterinarians and trust their judgment (I was rebuffed when I tried making suggestions and predictions*, *so I mostly disengaged after the first bill was passed)*.*”* (KI 1, CA/MD)

On another note, multiple KIs expressed that veterinarians are more involved in the administration and dispensation of antimicrobials than before the bill was passed and are more cognizant of producers’ practices.

*“I think that you know vets are more engaged now*. *I mean there are producers who didn’t think that they needed a veterinarian before*, *and now they kind of don’t have as much of a choice*.*”* (KI 2, CA)

Because the role of veterinarians has been more clearly laid out in the bill, some KIs in the veterinary field expressed that they have more authority over how livestock should be cared for and how antimicrobials should be used (KIs 2, 8, 13).

#### Level of government at which antimicrobial use policy should be enacted

With a range of KIs providing information on city, state, and federal antimicrobial use policies, a recurring question became whether a subnational or national approach to regulation will be most effective moving forward. The SF Ordinance, the only legislation of its kind, emphasizes the specific aim of increasing consumer transparency while they purchase meat products, allowing them to “vote with their fork” (KI 16, SF). SF Ordinance respondents also note the potential national impact of local legislation:

*“One of the things that is distinct about this*, *and one of the things we were excited about doing this is because we saw this is more than local in scope*, *because we’re talking about national chains*, *because we’re talking about national brands*, *the information generated here can be very powerful*, *just even beyond this city’s borders*. *And I think there’s been a little bit of a failure to understand that at times*.*”* (KI 19, SF)

Other respondents didn’t support the effectiveness of this municipal-level legislation, commenting:

*“It makes no difference*. *It inappropriately targets large suppliers*, *of which some large suppliers would have a harder time reporting that information [antimicrobial use] because they have a more diverse supply stream*.*”* (KI 8, SF)

Opinions varied on whether state departments of agriculture or the FDA should pass future antimicrobial use legislation. Those who advocated for the success of state-level policies saw it as either (a) a stopgap measure or (b) the pressure necessary to effect change at a national level. Regarding the first perspective, KI 19 notes:

*“Antibiotic resistance is an urgent and constantly emerging threat to public health*, *and if local and state level regulation can sort of be a stopgap measure to reduce overuse or if they can release regulation that is more targeted at certain types of overuse*, *then certainly there’s room for progress to be made at state levels*.*”* (KI 19, SF)

To the second point, advocates for state-level antimicrobial use policies argue that more stringent federal action won’t be taken until enough pressure has been placed by many states:

*“I tend to think the way things are going to work is that we’re going to make progress in the States first and eventually we’ll be able to demonstrate that*, *despite all the challenges and then there will be pressure on the federal government*, *and then we’ll eventually do things at the federal level*.*”* (KI 3, CA)

*“You get to a tipping point where all of a sudden there’s enough differences going on in different states …that the groups that have been opposed to what you’re doing say*, *we just need a federal policy*, *we don’t want to deal with 50 different sets of regulations*.*”* (KI 1, CA)

Those that think a federal policy would be more effective than a state approach cite the politicization of health and environmental issues in the United States as a main reason:

*“I feel that while this issue isn’t one that should be politicized it is…I have to imagine what you found in interviewing the California people*, *it’s a good law*, *but Maryland’s is better*. *But they’ve done nothing in California*, *because the California Agriculture Department hasn’t defined what routine use is*. *We don’t want 50 of those plus the territories going on*. *So it makes sense that the federal government determines*, *figures out a plan and then reports the information*.*”* (KI 15, SF)

Others echoed sentiments that a national surveillance system would be more efficient and eliminate loopholes created by sub-national policies:

*“I think it just has to be a national effort and not a state-by-state effort*. *Because there’s*, *it’s too piecemeal and too easy to go around the current reg [regulation]*. *I think the FDA is on the right track*, *but they’re government*, *federal government*, *so they’re going to be slow*. *And that’s unfortunate*, *but*, *but I think they’re on the right track*. *I don’t think they’re there yet*.*”* (KI 6, CA)

Several KIs mentioned FDA plans to enact a policy similar to Maryland’s or California’s within five years but note that COVID-19 has slowed these plans (KI 6, KI 8). No such information has yet been released by the FDA.

### Recommendations

Finally, we synthesized the key informant perspectives above into a list of recommendations for future antimicrobial use legislation in the animal agriculture sector (Table 3). To facilitate policymakers’ incorporation of these findings into future antimicrobial use policy at the subnational level, we provide recommendations for each step of the legislative process (writing, passing, and implementation).

**Table 3:** Summary of key informants’ (KI) recommendations for future antimicrobial use policy.

Category	Recommendation
Writing policy	• Use precise language in policy writing, to minimize key elements being left up to interpretation (KIs 1, 3, 5, 7, 17) • Define key concepts including ‘medically important antimicrobials’ and ‘routine uses’ of antimicrobials, as defined by the FDA (KIs 3, 7, 15) [13, 14]. • The intent to use antimicrobials to treat infections, rather than prevent disease, should be made clear in all policies (KI 2, 3, 19) • Policies should clearly designate which government entity and/or individuals are responsible for antimicrobial use data collection, analysis, and reporting, to ensure these processes are actually implemented Delegation of roles should be stated in a coherent manner, to ensure efficient implementation, as well as procedures for reporting data (KI 5, 7, 13) • Funding should be allocated for each section of the bill, with special attention to data analysis and reporting (KI 13, 15, 18) • Identify potential loopholes that would enable producer noncompliance (KIs 1, 5, 6, 8, 10, 12, 19)
Passing policy	• Garner consumer support of antimicrobial-free production—as observed in Maryland, this places pressure on food animal producers to adhere to antimicrobial use policies (KIs 1, 6) • Include diverse stakeholder groups in policy making process to increase support (e.g., veterinarians, producers, and the nonprofit sector) (KIs 1, 2, 5, 11)
Implementing policy	• Standardize formats for reporting use of medically important antimicrobials, to address logistical challenges of missing data or incomplete Veterinary Feed Directives (KIs 13, 19) • Incorporate management of an antimicrobial use database into implementation of policy (example being the use of Global VetLink in Maryland) (KIs 5, 14, 15, 17, 18).

## Discussion

The purpose of this study was to investigate the implementation and impact of three sub-national policies regarding antimicrobial use and transparency in the U.S. agricultural sector. Findings from key informant interviews fell into four main themes, which frequently overlapped in key informant interviews. Assessing them in conjunction, we developed two key considerations for future legislation: 1) a need to determine which legislative level future antimicrobial use policies may be most effective, and 2) a need to provide a set of recommendations for writing, passing, and implementing future antimicrobial use policies in the agricultural sector.

### City, state, or federal policy?

KI’s differed in opinion on whether city, and state, federal policy and/or a combination could be most effective in promoting judicious use of antimicrobials in the agricultural sector. However, the conclusion can be made that each legislative level serves a unique purpose, which KIs agreed upon, and both city- and state-level policies place pressure on the federal government, including the FDA, and national animal production companies to manage antimicrobial use more effectively.

At the city level, the SF Ordinance sought to increase transparency, providing information via reports to consumers about how much, when, and why antimicrobials were being used in the meat or poultry they purchased and consumed [35, 39]. All San Francisco KIs agreed that giving consumers the information to ‘vote with their fork’ was a main goal of the city’s policy. Due to the consolidation of the supermarket sector, grocery chains now subject to the SF policy’s reporting requirements within the city may make changes to their antimicrobial use reporting across branches, at the national or global scale, a key strength of San Francisco’s city level approach [40]. The SF Ordinance has been a model for policy in other states as well: Seattle wrote a similar policy shortly after San Francisco enacted Ordinance 204–17, but the enactment has been slowed due to COVID-19. The challenge with the Ordinance, identified in interviews, is that grocers and producers are jointly held financially responsible when producers don’t report the required antimicrobial use data to the Department of the Environment, which many producers aren’t yet doing. A recent review finds the rates of reporting from grocers in San Francisco have remained low; during the last two years, grocers only reported antimicrobial use from one of 29 beef products, and one of 18 pork products [40]. This is indication that policy implementation, with regards to increasing both producer and grocer reporting of antimicrobials in meat products, hasn’t been entirely successful.

The argument for state level regulation of antimicrobial use had multiple perspectives. First, KIs raised the argument that state agricultural sectors vary widely, and so may the policies that best serve them. For example, Texas, the state leading sales of cattle and calves in the U.S. ($13 billion) may require different antimicrobial stipulations than Georgia, which leads broiler production with 1.33 billion chickens per year [41, 42]. The second argument identified in this study is that many states might need to enact policies before reaching a “tipping point” where the FDA takes action to institute stricter antimicrobial use requirements. New York and Illinois, both with similar antimicrobial use policies on hold in committee, may add to the state-level legislation that puts pressure on the FDA to pass similar legislation [29, 31]. However, as revealed in interviews, the problem with the state-level approach to regulation is that if each state has different antimicrobial use requirements, producers may find them “too piecemeal, too easy to go around.”

The majority opinion of KIs in this study was that federal regulation of antimicrobials in the agriculture sector would be the most effective way to increase judicious use in the long term. The FDA shared in their 2020 Summary Report that though sales of medically important antimicrobials for use in food-producing animals decreased 3% from 2019 to 2020 and 38% since 2015, the significant decrease in sales occurred after GFI #263, passed in 2017 [14, 43].

### Antimicrobial use data collection & reporting

On the whole, KIs also agreed that a national surveillance system for sales and use of medically important antimicrobials would provide important data for environmental surveillance of emerging AMR. KIs emphasized that reporting use of medically important antimicrobials in livestock needs improvement in both the CA and MD policies, although Maryland has made significantly more progress than California in this regard. As mentioned, the second version of the MD policy made these requirements clearer, although this study also revealed logistical challenges with data analysis, including missing data, incomplete VFDs, and the lack of enforcement for accurate and complete data reporting.

Additionally, as made clear by KIs, while the FDA requests specific AMU data in VFDs, they do not mandate a single standardized format for reporting. Despite recommendations by the Government Accountability Office, currently no federal agency mandates farms to report AMU [44]. As a result, data on antimicrobial sales for food-producing animals is the only current available proxy. Through their sub-national policies, both Maryland and San Francisco have sought to address this paucity of data. Maryland Department of Agriculture’s partnership with Global VetLink uses VFDs to collect antimicrobial use data without having to contact each producer individually [17]. After the SF Ordinance passed, a database was built to collect information of antimicrobial use in hopes of easing the process for the next city who tries to pass similar legislation. This effort has been less than successful, however, in both 2019 and 2020, only 2% of beef and pork producers reported antibiotic use data to the city [39, 45]. Interviews in this study reinforced this finding; respondents mentioned the tendency that large meat producers have towards secrecy, and the fact that many of them band together to reinforce this opacity.

Models do exist for national data reporting on agricultural antimicrobial use. The European Union and United Kingdom have set examples of such surveillance systems. Initiated by the European Medicines Agency in 2009, the European Surveillance of Veterinary Antimicrobial Consumption (ESVAC) project collects and shares data on antimicrobial sales and use for food-animals across 31 countries [46]. Also of note, a 52% decline in antimicrobial sales in the UK is attributed to the Responsible Use of Medicines in Agriculture Alliance of 2017 [18]. In Denmark, the Danish Program for surveillance of antimicrobial consumption and resistance (DANMAP) in bacteria from food animals, food, and humans, began in the 2000s across Denmark [47]. The key to DANMAP’s success in reducing AMR in Denmark lies in the collaborative nature of the effort, with stakeholders involved in enactment at all levels of the “farm-to-fork food chain” [47]. DANMAP put AMR on political, agriculture, and public health sector agendas, bringing about awareness and change stemming from a united interest in acting to prevent the threat of a global health crisis. With a federal policy that incorporated national antimicrobial use surveillance into its implementation, the U.S. too could employ improved legislation to prevent the spread of AMR.

### Considerations in writing, passing, and implementing future antimicrobial use policy

It was emphasized by KIs that, due to the political negotiation process, the way the California policy was written left many key aspects of the policy up to interpretation, such as the way antimicrobials could legally be used and the reporting requirements of antimicrobial use data. In comparison, Maryland’s policy was more successful in creating clear requirements and definitions. This was primarily accomplished through passing the second version of the bill clarifying language on data collection and reporting. The amended version of Maryland’s bill, SB471/HB652, added and clarified definitions for “administered in a regular pattern,” “control of the spread of disease or infection,” “elevated risk,” “prophylaxis,” and “treating a disease or infection” [27]. Additionally, multiple KIs explained that in California, the bill had been interpreted as leniently as possible by some groups, including the CDFA. Future policies should be written in a way that makes them easy and clear to enforce and implement.

Policymakers writing new antimicrobial use policy in the United States can also look to progress made in Europe as example. In contrast to the FDA and sub-national policy in the U.S., the European Parliament enacted a ban on antimicrobial use for disease prevention or growth promotion and established an obligation for member states to collect data on sales and use of antimicrobials, effective on January 28, 2022 [40, 48]. Examples of successful and substantive reductions of antimicrobial use in agriculture have been seen in The Netherlands, Denmark, and elsewhere [7, 49].

KIs concurred widely that regardless of policy specifics, stakeholder perspectives and attitudes heavily influence whether an antimicrobial use policy will in practice reduce inappropriate antimicrobial use in animal agriculture. The importance of stakeholder perspectives in antimicrobial use policy has also been supported previously in qualitative analyses of SB-27 [50]. Large animal producers have opposed antimicrobial use policies in San Francisco and California in particular, but the situation differed in Maryland. Consumer preferences placed the pressure on corporations to change their practices; several restaurant chains committed to reducing antimicrobial use, and large chicken producers have also begun shifting towards antimicrobial-free production, including the company Perdue in 2016 [9, 28, 51]. Additionally, research based on a large study by Perdue found that the benefits of using antibiotics in broiler production were insufficient to offset the cost of the antibiotics [52]. Once the company reduced their antimicrobial use, they pivoted to take advantage of the change and began marketing their poultry as ‘no antibiotics ever—the Perdue way,’ so when Maryland passed the Keep Antibiotics Effective Act, there was less producer resistance [44].

This highlights the role of industry in decision-making regarding reducing antimicrobial use and supporting city and state policies [9]. When stakeholders are supportive, antimicrobial use policies face less pushback. The CA policy, in comparison to MD, faced larger opposition from the Cattlemen’s Association, large animal producers, and the Department of Agriculture, challenging the policy’s approval and implementation. In addition, when these stakeholders are not supportive, KIs warned that producers often find loopholes to antimicrobial use policies. Though the hope is for legislation to be a productive means for change, this finding demonstrates the leverage industrial agriculture holds in the United States, and therefore the importance of attaining these stakeholder’s cooperation in a policy’s success.

While city and state-level policies have attempted to address some of the lack of federal oversight on antimicrobial use in the food-animal sector in the United States, this study identified barriers to the success in both regulation and reporting they desired to achieve. Recommendations for achieving better regulation and reporting include standardizing AMU reporting formats across states, garnering consumer support for antibiotic-free food-animal production and using exceedingly concise language in writing policy so as to avoid loopholes and noncompliance. Most importantly, key informants highlighted the need for gaining diverse stakeholder support–veterinarians, advocacy groups and policymakers, for example–as critical to the success of both passing and implementing AMU policy.

### Study limitations

To the authors’ knowledge, this is the first qualitative study of professionals, with ranging topical expertise, on all existing antimicrobial use policies in the United States, providing insights and recommendations for future legislation to curb AMR through the agriculture sector. However, the study has limitations. First, this was a small qualitative study, with only 19 KIs. Inclusion of a larger number of KIs could have led to the identification of additional themes, or different points of consensus on the importance of the ideas raised.

Second, KIs were selected based on subject-area expertise known to the study team and may not fully represent all informants who could have added other perspectives. The study team intentionally interviewed both KIs involved in the writing and passing of the bill, and KIs whose work was impacted by the passing of the bill, but who were not privy to ‘insider’ information for each of the three policies. However, based on response to our requests, there were not an equal distribution of interviewees across the different policies.

Third, as the policies analyzed have all been enacted in the past three years, the majority of KIs mentioned that conclusive evidence for the legislation’s role in reduction of antimicrobial use or AMR is not yet available, or it was “too soon to tell” the true impact of each bill. We also focused on three sub-national policies, but as many KIs mentioned, federal policy is the most important path forward, and future studies may wish to discuss it in greater depth with KIs from high-production states such as Georgia, Iowa, and North Carolina.

## Conclusion

This study provides important considerations for legislators aiming to reduce antimicrobial use and increase the transparency of use in U.S. food-animal production. Our study provides qualitative support for the importance of sub-national antimicrobial use policies, but mainly as a means to urge stricter antimicrobial use regulation at the federal level. As an increasing number of cities and states move towards limiting the use of antimicrobials in food-animals for treatment purposes only, key considerations for future policies should include: 1) precise definitions of antimicrobial use regulation; 2) clearly defined funding streams to ensure proper data collection and reporting; and 3) inclusion of diverse stakeholders in policy development and implementation, which could support the impact of the policy. Antimicrobial use in agriculture has been shown to be an important driver in the spread of AMR. Understanding the effects of antimicrobial use policies will be essential to determining how each policy affects actual antimicrobial use in food-animal production both regionally and over time.

## Supporting information

S1 File Semi-structured interview guides for 1) California, 2) Maryland, and 3) San Francisco policy interviews. (DOCX)

S1 Table Qualitative codebook from axial coding of semi-structured stakeholder interviews. (DOCX)

## References

[1] O’Neill J. Tackling drug-resistant infections globally: final report and recommendations. Government of the United Kingdom; 2016 May [cited 2022 Feb 18]. Available from: https://apo.org.au/node/63983

[2] Alvarez-Uria G, Gandra S, Mandal S, Laxminarayan R. Global forecast of antimicrobial resistance in invasive isolates of Escherichia coli and Klebsiella pneumoniae. Int J Infect Dis. 2018 Mar;68:50–3. doi: 10.1016/j.ijid.2018.01.011 29410253 PMC5889426

[3] Burnham JP, Olsen MA, Kollef MH. Re-estimating annual deaths due to multidrug-resistant organism infections. Infect Control Hosp Epidemiol. 2019 Jan;40(1):112–3. doi: 10.1017/ice.2018.304 30463634 PMC6602528

[4] Dadgostar P. Antimicrobial Resistance: Implications and Costs. Infect Drug Resist. 2019 Dec 20;12:3903–10. doi: 10.2147/IDR.S234610 31908502 PMC6929930

[5] 2015 Summary Report on Antimicrobials Sold or Distributed for Use in Food-Producing Animals.: 58.

[6] Murray CJ, Ikuta KS, Sharara F, Swetschinski L, Aguilar GR, Gray A, et al. Global burden of bacterial antimicrobial resistance in 2019: a systematic analysis. The Lancet. 2022 Feb 12;399(10325):629–55. 10.1016/S0140-6736(21)02724-0 PMC8841637 35065702

[7] Levy SB, FitzGerald GB, Macone AB. Changes in Intestinal Flora of Farm Personnel after Introduction of a Tetracycline-Supplemented Feed on a Farm. New England Journal of Medicine. 1976 Sep 9;295(11):583–8. doi: 10.1056/NEJM197609092951103 950974

[8] Nordstrom L, Liu C, Price L. Foodborne urinary tract infections: a new paradigm for antimicrobial-resistant foodborne illness. Frontiers in Microbiology. 2013 [cited 2022 Jan 17];4. Available from: https://www.frontiersin.org/article/10.3389/fmicb.2013.00029 23508293 10.3389/fmicb.2013.00029 PMC3589730

[9] Silbergeld EK, Graham J, Price LB. Industrial Food Animal Production, Antimicrobial Resistance, and Human Health. Annual Review of Public Health. 2008;29(1):151–69. doi: 10.1146/annurev.publhealth.29.020907.090904 18348709

[10] Smalla K, Cook K, Djordjevic SP, Klümper U, Gillings M. Environmental dimensions of antibiotic resistance: assessment of basic science gaps. FEMS Microbiology Ecology. 2018 Dec 1;94(12):fiy195. doi: 10.1093/femsec/fiy195 30277517

[11] Taylor P, Reeder R. Antibiotic use on crops in low and middle-income countries based on recommendations made by agricultural advisors. CABI Agriculture and Bioscience. 2020 Jun 23;1(1):1.

[12] Manyi-Loh C, Mamphweli S, Meyer E, Okoh A. Antibiotic Use in Agriculture and Its Consequential Resistance in Environmental Sources: Potential Public Health Implications. Molecules. 2018 Mar 30;23(4):795. doi: 10.3390/molecules23040795 29601469 PMC6017557

[13] Wallinga D MD, Kar A. New Data: Animal vs. Human Antibiotic Use Remains Lopsided. NRDC. [cited 2022 Jan 17]. Available from: https://www.nrdc.org/experts/david-wallinga-md/most-human-antibiotics-still-going-us-meat-production

[14] 2020 Summary Report On Antimicrobials Sold or Distributed for Use in Food-Producing Animals.: 49.

[15] Muloi D, Fèvre EM, Bettridge J, Rono R, Ong’are D, Hassell JM, et al. A cross-sectional survey of practices and knowledge among antibiotic retailers in Nairobi, Kenya. J Glob Health. [cited 2020 Sep 29];9(2). Available from: https://www.ncbi.nlm.nih.gov/pmc/articles/PMC6708591/10.7189/jogh.09.020412 PMC6708591 31489183

[16] Tang KL, Caffrey NP, Nóbrega DB, Cork SC, Ronksley PE, Barkema HW, et al. Restricting the use of antibiotics in food-producing animals and its associations with antibiotic resistance in food-producing animals and human beings: a systematic review and meta-analysis. The Lancet Planetary Health. 2017 Nov 1;1(8):e316–27. doi: 10.1016/S2542-5196(17)30141-9 29387833 PMC5785333

[17] National Antimicrobial Resistance Monitoring System for Enteric Bacteria (NARMS) | NARMS | CDC. 2020 [cited 2022 Jan 13]. Available from: https://www.cdc.gov/narms/index.html

[18] Borriello P. UK veterinary antibiotics sales more than halved over the past six years. GOV.UK. [cited 2022 Jan 13]. Available from: https://www.gov.uk/government/news/uk-veterinary-antibiotics-sales-more-than-halved-over-the-past-six-years

[19] Antibacterial agents in clinical development: an analysis of the antibacterial clinical development pipeline, including tuberculosis. World Health Organization; 2017. Available from: https://apps.who.int/iris/handle/10665/258965.

[20] Collignon PJ, McEwen SA. One Health—Its Importance in Helping to Better Control Antimicrobial Resistance. Tropical Medicine and Infectious Disease. 2019 Mar;4(1):22. doi: 10.3390/tropicalmed4010022 30700019 PMC6473376

[21] Guidance for Industry #213: New Animal Drugs and New Animal Drug Combination Products Administered in or on Medicated Feed or Drinking Water of FoodProducing Animals: Recommendations for Drug Sponsors for Voluntarily Aligning Product Use Conditions with GFI #209. 213 Dec, 2013. Available from: https://www.fda.gov/media/83488/download

[22] Sneeringer S, Short G, MacLachlan M, Bowman M. Impacts on Livestock Producers and Veterinarians of FDA Policies on Use of Medically Important Antibiotics in Food Animal Production. Applied Economic Perspectives and Policy. 2020;42(4):674–94.

[23] Hoelzer K. Judicious Animal Antibiotic Use Requires Changes to Drug Labels. [cited 2022 Jan 17]. Available from: http://pew.org/2dcGrQF

[24] Gubrium JF, Holstein JA, Marvasti AB, McKinney KD. The SAGE Handbook of Interview Research: The Complexity of the Craft. SAGE; 2012. 625 p.

[25] Guidos R J. Combating Antimicrobial Resistance: Policy Recommendations to Save Lives. Clin Infect Dis. 2011 May 1;52(Suppl 5):S397–428. doi: 10.1093/cid/cir153 21474585 PMC3738230

[26] Senate Bill No. 27: An act to add Chapter 4.5 (commencing with Section 14400) to Division 7 of the Food and Agricultural Code, relating to livestock. SB-27 Oct 10, 2015. Available from: https://leginfo.legislature.ca.gov/faces/billTextClient.xhtml?bill_id=201520160SB27

[27] Wellington M. Maryland’s updated “Keep Antibiotics Effective Act” becomes law | U.S. PIRG. U.S. Public Interest Research Group. [cited 2022 Jan 17]. Available from: https://uspirg.org/news/usp/maryland%E2%80%99s-updated-%E2%80%9Ckeep-antibiotics-effective-act%E2%80%9D-becomes-law

[28] Meyer Z. Tyson Foods will eliminate antibiotics in chicken. USA Today. 2017 [cited 2022 Jan 17]. Available from: https://www.usatoday.com/story/money/business/2017/05/01/poultry-giant-tyson-boot-antibiotics-chicken/100970854/

[29] Cullerton J, Hunter M. IDPH-ANTIBIOTICS-ANIMALS. SB3429 Jan 9, 2019. Available from: https://www.ilga.gov/legislation/BillStatus.asp?DocNum=3429&GAID=14&DocTypeID=SB&SessionID=91&GA=100

[30] Leach D. An Act prohibiting the administration of certain antimicrobial agents in agriculture; providing for inspection and testing of agricultural operations, for enforcement, for reporting by agricultural operations and for alternatives to administration of antimicrobial agents to animals; and making related repeals. SB 246 Jan 27, 2017. Available from: https://www.legis.state.pa.us/CFDOCS/billInfo/billInfo.cfm?syear=2017&sInd=0&body=S&type=B&bn=246

[31] Kavanagh B. Senate Bill S3115: Relates to the non-therapeutic use of antimicrobial agents in animals. S 3115 2022. Available from: https://www.nysenate.gov/legislation/bills/2021/S3115

[32] Hayward S. Relating to protecting antibiotics for human public health; declaring an emergency. SB 920A 2015. Available from: https://olis.oregonlegislature.gov/liz/2015R1/Measures/Overview/SB920

[33] Senate Bill 422—Keep Antibiotics Effective Act of 2017. SB-422 May 27, 2017. Available from: https://legiscan.com/MD/bill/SB422/2017

[34] Senate Bill 471: Agriculture—Use of Antimicrobial Drugs—Limitations and Reporting Requirements. SB-471 May 25, 2019. Available from: https://legiscan.com/MD/text/SB471/2019

[35] Ordinance amending the Environment Code to require certain retailers of raw meat and poultry to report the use of antibiotics in such products to the Department of the Environment, and require City departments to report the use of antibiotics in raw meat and poultry purchased by the City to the Department of the Environment. 204–17 Oct 3, 2017. Available from: https://sfgov.legistar.com/View.ashx?M=F&ID=5527122&GUID=416E70B6-7805-4869-8784-B5D8BA8A043B

[36] ATLAS.ti Scientific Software Development GmbH [ATLAS.ti 22 Mac]. ATLAS.ti. [cited 2022 Sep 12]. Available from: https://atlasti.com

[37] O’Connor C, Joffe H. Intercoder Reliability in Qualitative Research: Debates and Practical Guidelines. International Journal of Qualitative Methods. 2020 Jan 1;19:1609406919899220.

[38] Annual Summary Report: Veterinary Feed Directives 2019. California Department of Food & Agriculture; 2019. Available from: https://www.cdfa.ca.gov/is/ffldrs/pdfs/AUS_VFD_Summary_Report_2019.pdf

[39] Weber A, Jackson J, Monnet J, Raphael D, Sheehan C. San Francisco Antibiotic Use in Food Animals Ordinance Reporting Year 2019. SF Environment; 2020. Available from: https://sfenvironment.org/sites/default/files/sf_antibiotic_use_in_food_animals_ordinance_2019_annual_report.pdf

[40] Wallinga D, Smit LAM, Davis MF, Casey JA, Nachman KE. A Review of the Effectiveness of Current US Policies on Antimicrobial Use in Meat and Poultry Production. Curr Environ Health Rep. 2022;9(2):339–54. doi: 10.1007/s40572-022-00351-x 35477845 PMC9090690

[41] Broilers: Inventory by State, US. USDA—National Agricultural Statistics Service—Charts and Maps. 2020 [cited 2022 Jan 13]. Available from: https://www.nass.usda.gov/Charts_and_Maps/Poultry/brlmap.php

[42] Cattle Industry. USDA—National Agriculture Statistics Service; 2015. Available from: https://www.nass.usda.gov/Publications/Highlights/2015/Cattle_Highlights.pdf

[43] Dall C. FDA report shows small decline in antibiotic use on farms. CIDRAP. [cited 2022 Jan 13]. Available from: https://www.cidrap.umn.edu/news-perspective/2021/12/fda-report-shows-small-decline-antibiotic-use-farms

[44] Antibiotic resistance: more information needed to oversee use of medically important drugs in food animals. United States Government Accountability Office; 2017. Available from: https://www.gao.gov/assets/690/683130.pdf

[45] San Francisco Antibiotic Use in Food Animals Ordinance Reporting Year 2018. SF Environment; 2020. Available from: https://sfenvironment.org/sites/default/files/fliers/files/sf_antibiotic_use_in_food_animals_ordinance_2018_reporting_year_annual_report_march_2020.pdf

[46] EMA. European Surveillance of Veterinary Antimicrobial Consumption (ESVAC). European Medicines Agency. 2018 [cited 2022 Nov 11]. Available from: https://www.ema.europa.eu/en/veterinary-regulatory/overview/antimicrobial-resistance/european-surveillance-veterinary-antimicrobial-consumption-esvac

[47] Wielinga PR, Jensen VF, Aarestrup FM, Schlundt J. Evidence-based policy for controlling antimicrobial resistance in the food chain in Denmark. Food Control. 2014 Jun 1;40:185–92.

[48] More SJ. European perspectives on efforts to reduce antimicrobial usage in food animal production. Irish Veterinary Journal. 2020 Jan 27;73(1):2. doi: 10.1186/s13620-019-0154-4 32002180 PMC6986017

[49] Speksnijder DC, Mevius DJ, Bruschke CJM, Wagenaar JA. Reduction of Veterinary Antimicrobial Use in the Netherlands. The Dutch Success Model. Zoonoses and Public Health. 2015;62(s1):79–87. 25421382 10.1111/zph.12167

[50] Innes GK, Markos A, Dalton KR, Gould CA, Nachman KE, Fanzo J, et al. How animal agriculture stakeholders define, perceive, and are impacted by antimicrobial resistance: challenging the Wellcome Trust’s Reframing Resistance principles. Agric Human Values. 2021 Dec;38(4):893–909. doi: 10.1007/s10460-021-10197-y 34776605 PMC8588841

[51] Bunge J. Perdue Farms Eliminated Antibiotics From Chicken Supply. Wall Street Journal. 2016 Oct 6 [cited 2022 Mar 24]; Available from: http://www.wsj.com/articles/perdue-farms-eliminated-all-antibiotics-from-its-chicken-supply-1475775456

[52] Graham JP, Boland JJ, Silbergeld E. Growth Promoting Antibiotics in Food Animal Production: An Economic Analysis. Public Health Rep. 2007 Jan 1;122(1):79–87. doi: 10.1177/003335490712200111 17236612 PMC1804117

