# Supplementary material for: Weaning U.S. food-animals off antimicrobials: What can we learn from state- and city-level policies?
Source: PLoS One. 2023 Mar 15;18(3):e0282315. doi: 10.1371/journal.pone.0282315 (PMC10016712; doi:10.1371/journal.pone.0282315)
Supplement: S1 File — (DOCX) [file pone.0282315.s001.docx]

**S1: Semi-structured interview guides for 1) California, 2) Maryland, and 3) San Francisco policy interviews.**

**1) Semi-structured interview guide for California (SB 27)**

Hello __________, thank you for agreeing to speak to us about SB 27. We really appreciate it. We have some questions that we would like to ask you about SB 27 so please share whatever you are able. I am first going to summarize SB 27 to make sure that I understand the basic goals of the legislation, and it would be great if you can highlight anything that I’ve missed about the bill.

Our understanding of SB 27 is that it aims to reduce the overuse and misuse of medically important antibiotics by only allowing veterinarians who have a valid veterinarian-client-patient relationship (VCPR) to administer, dispense, or prescribe medically important antimicrobial drugs for therapeutic purposes such as to treat a disease or infection, control the spread of a disease or infection, and NOT for the purposes of promoting weight gain or improving feed efficiency. Medications can also be dispensed by a licensed pharmacy and veterinary food animal drug retailers which are only used for livestock producers with seller’s permits. Prescriptions for medically important antibiotics may not go longer than a year and veterinarians must also complete a minimum of one credit hour of continuing education on the judicious use of medically important antibiotics every four years as a condition of licensure. The Department of Food and Agriculture may request and receive copies of veterinary feed directives (VFD) and is required to gather information on medically important antibiotics sales and usage, antibiotic resistant bacteria, and livestock management practice data.

1. In your view, have we summarized the main aspects of what this bill does?
2. Can you briefly describe how you have become familiar with SB 27?
3. On a scale from 0 to 10, 0 being completely ineffective and 10 being completely effective, did SB 27:
   1. Restrict/ban antibiotic usage for non-therapeutic purposes?
   2. Limit who can administer, dispense, or prescribe medically important antibiotics to livestock?
   3. Set a reasonable duration limit on the use of medically important antibiotics?
   4. Request important data concerning antibiotics sales and usage and livestock management practice?
   5. Make an impact in curbing the misuse and overuse of medically important antibiotics in livestock in Illinois?
4. Based on your current knowledge, what positive/negative effects resulted from the implementation of SB 27?
   1. Was it beneficial for some groups and detrimental for others?
5. What were the main challenges you observed during the implementation of SB 27 and after?
   1. Were there groups/individuals/organization etc. either at the state or national level that helped facilitate or hinder the implementation of this legislation?
   2. What elements helped make the implementation of SB 27 successful?
6. If another state were to implement a similar legislation, what would you recommend they take into consideration to make their legislation effective?
7. Are there any other points that you would like for people to know about SB 27?
8. Are there other individuals you recommend we talk to regarding this legislation or others like it?

**2)** **Semi-structured interview guide for SB471/HB652 in Maryland**

Hello __________, thank you for agreeing to speak to us about SB471/HB652. We really appreciate it. We have six questions that we would like to ask you about SB471/HB652 and please share whatever you are able. I’m first going to summarize SB471/HB652 to make sure that I understand the basic goals of the legislation, and it would be great if you can highlight anything that I’ve missed about the bill.

Our understanding of SB471/HB652 is that it aims to reduce the overuse and misuse of medically important antibiotics by requiring reporting of antibiotic usage on large farms to ensure compliance and track progress. It also aims to restrict use of antibiotics on livestock and swine in good health and adds exemptions for dairy farms and other small farms who have a herd size fewer than 300. Veterinarians are required to report annual use of medically important antibiotics.

1. In your view, have we summarized the main aspects of what this bill does?
2. Can you briefly describe how you have become familiar with SB471/HB652?
3. On a scale from 0 to 10, 0 being completely ineffective and 10 being completely effective, did SB471/HB652:
   1. Restrict/ban antibiotic usage for non-therapeutic purposes?
   2. Limit who can administer, dispense, or prescribe medically important antibiotics to livestock?
   3. Set a reasonable duration limit on the use of medically important antibiotics?
   4. Request important data concerning antibiotics sales and usage and livestock management practice?
   5. Make an impact in curbing the misuse and overuse of medically important antibiotics in livestock in Illinois?
4. Based on your current knowledge, what positive/negative effects resulted from the implementation of SB471/HB652?
   1. Was it beneficial for some groups and detrimental for others?
5. What were the main challenges you observed during the implementation of SB471/HB652 and after?
   1. Were there groups/individuals/organization etc. either at the state or national level that helped facilitate or hinder the implementation of this legislation?
   2. What elements helped make the implementation of SB471/HB652 successful?
6. If another state were to implement a similar legislation, what would you recommend they take into consideration to make their legislation effective?
7. Are there any other points that you would like for people to know about SB471/HB652?
8. Are there other individuals you recommend we talk to regarding this legislation or others like it?

**3) Semi-structured interview guide for San Francisco Ordinance No. 204-17**

Hello __________, thank you for agreeing to speak to us about San Francisco Ordinance No. 204-17. We really appreciate it. We have some questions that we would like to ask you about the ordinance and please share whatever you are able. I’m first going to summarize San Francisco Ordinance No. 204-17 to make sure that I understand the basic goals of the policy, and it would be great if you can highlight anything that I’ve missed about the ordinance.

Our understanding of San Francisco Ordinance No. 204-17 is that it aims to reduce the overuse and misuse of medically important antibiotics by requiring grocery stores chains which have at least 25 locations and have at least one location in San Francisco, as well as for any City Department which purchases raw meat, to report any antibiotics used in meat products to their customers. A report must include the reason for antibiotic use, whether it is certified by a third-party (unaffiliated with the grocer or producer such as the USDA), the number of animals, the amount of antibiotics used and whether the antibiotics used are medically important. Failure to file a report can led to fines, loss of permit, or jail time, and can justify lawsuits against the violator.

1. In your view, have we summarized the main aspects of what this policy does?
2. Can you briefly describe how you have become familiar with ordinance No. 204-17?
3. On a scale from 0 to 10, 0 being completely ineffective and 10 being completely effective, did ordinance No. 204-17:
   1. Subject grocers to report their meat and poultry producers’ antibiotics use policies and practices to the Department of the Environment yearly?
   2. Make an impact in curbing the misuse and overuse of medically important antibiotics in livestock by encouraging transparency between grocers and consumers on the use of antibiotics in meat and poultry products in San Francisco?
4. Based on your current knowledge, what positive/negative effects resulted from the implementation of ordinance No. 204-17?
   1. Was it beneficial for some groups and detrimental for others?
5. What were the main challenges you observed during the implementation of ordinance No. 204-17 and after?
   1. Were there groups/individuals/organization etc. either at the state or national level that helped facilitate or hinder the implementation of this policy?
   2. What elements helped make the implementation of ordinance No. 204-17 successful?
6. If another city were to implement a similar policy, what would you recommend they take into consideration to ensure their ordinance is effective?
7. Are there any other points that you would like for people to know about ordinance No. 204-17?
8. Are there other individuals you recommend we talk to regarding this policy or others like it?
