# Supplementary material for: Weaning U.S. food-animals off antimicrobials: What can we learn from state- and city-level policies?
Source: PLoS One. 2023 Mar 15;18(3):e0282315. doi: 10.1371/journal.pone.0282315 (PMC10016712; doi:10.1371/journal.pone.0282315)
Supplement: S1 Table — (DOCX) [file pone.0282315.s002.docx]

**S2 Table: Qualitative Codebook from Axial Coding of Semi-Structured Stakeholder Interviews**

| ● DATA.Challenges in data collection with existing bill |
| --- |
| ● DATA.Challenges with data reporting resulting from the bill |
| ● DATA.More stringent data reporting is a hindrance to passing bill |
| ● DATA.More stringent data reporting is necessary |
| ● DATA.Need more time to tell based on data if bill has been effective |
| ● F/S.Federal government should enforce |
| ● F/S.Need for a national antibiotic use surveillance system |
| ● F/S.Need to enact a lot of state policies to pressure a new federal policy |
| ● F/S.State governments should enforce |
| ● FUT.Advice: don't work with public interest groups |
| ● FUT.Advice: engage the public health community |
| ● FUT.Advice: work with farmers/producers |
| ● FUT.Advice: work with grocers |
| ● FUT.Advice: work with public interest groups |
| ● FUT.Consumer pressure helps with antibiotic regulation/reduction |
| ● FUT.COVID has slowed progress |
| ● FUT.Department of Agriculture pushback |
| ● FUT.Need for funding to enact bill |
| ● FUT.Need for government/political buy-in to pass bill |
| ● FUT.Pharmaceutical industry pushback |
| ● FUT.Veterinary Medical Association pushback |
| ● NEED.Goal is to increase transparency of antibiotic use reporting |
| ● NEED.Governor/political support |
| ● NEED.Need for clear language in bill on antibiotic use (duration, therapeutic, etc. |
| ● NEED.Need for explicit language/mandate in bill on data reporting |
| ● NEED.Need to get hospitals/medical community involved |
| ● NEED.Need to involve diverse stakeholders to enact bill |
| ● NEED.Producers should have to report antibiotic use, not grocers |
| ● NEG.Antibiotic regulation is an animal welfare issue |
| ● NEG.Bill did not effectively increase antibiotic use transparency |
| ● NEG.Bill didn't effectively limit dispensation of medically important antibiotics |
| ● NEG.Bill had no impact further than existing federal legislation |
| ● NEG.Impacts on farms from bill |
| ● NEG.Impacts on meat prices from bill |
| ● NEG.Impacts on vets from bill |
| ● NEG.Producers found loopholes around bill |
| ● POS. Positive impact on grocer engagement from bill |
| ● POS.Beneficial for veterinarians |
| ● POS.Bill compels Department of Agriculture to collect and analyze data |
| ● POS.Bill did collect data on antibiotic use |
| ● POS.Bill did not have negative impacts |
| ● POS.Bill effectively banned AB usage for non-therapeutic uses |
| ● POS.Bill effectively limited dispensation of medically important antibiotics |
| ● POS.Bill raised awareness of antibiotic use |
| ● POS.Bill was a model for future policy |
| ● POS.Bill was effective in increasing antibiotic use transparency |
| ● POS.Producers save money from judicious antibiotic use |
| ● PROD.Meat industry pushback |
| ● PROD.Poultry industry antibiotic ban was easier than for large animals |
| ● PROD.Producers (poultry) setting example of going antibiotic-free helped bill |
| ● REG.Adherance was motivated through fines to grocers |
| ● REG.Adherance was motivated through fines to veterinarians |
| ● REG.Need for a tracking and/or enforcement system |
